# Cytomegalovirus acquisition in infancy and the risk of tuberculosis disease in childhood: a longitudinal birth cohort study in Cape Town, South Africa

**DOI:** 10.1016/S2214-109X(21)00407-1

**Published:** 2021-11-16

**Authors:** Leonardo Martinez, Mark P Nicol, Catherine J Wedderburn, Attie Stadler, Maresa Botha, Lesley Workman, David M le Roux, Heather J Zar

**Affiliations:** a Department of Epidemiology, School of Public Health, Boston University, Boston, MA, USA; b Division of Infection and Immunity, School of Biomedical Sciences, University of Western Australia, Perth, WA, Australia; c Division of Medical Microbiology, University of Cape Town, Cape Town, South Africa; d Department of Paediatrics and Child Health, Red Cross War Memorial Children's Hospital and South African Medical Research Council Unit on Child and Adolescent Health, Cape Town, South Africa; e Department of Clinical Research, London School of Hygiene & Tropical Medicine, London, UK

## Abstract

**Background:**

The risk of tuberculosis disease after recent exposure is greatest before age 5 years; however, the mechanisms explaining this increased risk are not well elucidated. Acquisition of viral infections, such as cytomegalovirus, in early life might modulate the immune system. We aimed to evaluate the acquisition of cytomegalovirus infection in infancy and the development of tuberculosis disease in children.

**Methods:**

In this prospective, birth cohort study we enrolled pregnant women who were between 20 and 28 weeks of gestation attending antenatal care in Paarl, a periurban setting outside of Cape Town, South Africa. Participants were recruited from two clinics (TC Newman and Mbekweni). Infants were given Bacillus Calmette–Guérin vaccination at birth as per national policy. Nasopharyngeal swabs for cytomegalovirus detection using qPCR were done for infants at birth, age 3 and 6 weeks, and age 3, 6, 12, and 24 months. Children were prospectively followed up for tuberculosis disease until age 9 years using tuberculin skin testing, radiographic examinations, GeneXpert, and sputum testing. Tuberculin skin tests were done at the 6-month visit and then at age 12, 24, 36, 48, and 60 months, and at the time of lower respiratory tract infection. We compared tuberculosis disease incidence after age 1 year or after age 6 months in children with and without cytomegalovirus infection using Cox regression and hazard ratios (HRs) with 95% CIs.

**Findings:**

Between March 5, 2012, and March 31, 2015, 1225 pregnant women were recruited and enrolled in the birth cohort. 88 (7%) women were excluded because of loss to antenatal follow-up or pregnancy losses. Of 1143 livebirths, 68 (6%) mother–infant pairs were excluded. In total, 963 children were serially tested for cytomegalovirus (7186 cytomegalovirus measurements taken; median six tests per child, IQR 2–11). The prevalence of congenital cytomegalovirus at age younger than 3 weeks was 2% (18 of 816). Cytomegalovirus positivity increased continuously with age from 3% (27 of 825) by age 6 weeks to 21% (183 of 882) by 3 months, 35% (315 of 909) by 6 months, and 42% (390 of 933) by 12 months. Mother–infant pairs were followed up for a median of 6·9 years (IQR 6·0–7·8). The risk of tuberculosis disease in children after age 1 year was higher in those with cytomegalovirus infection by age 6 weeks (adjusted HR 4·1, 95% CI 1·2–13·8; p=0·022), 3 months (2·8, 1·4–5·8; p=0·0040), 6 months (3·6, 1·7–7·3; p<0·0001), 12 months (3·2, 1·6–6·4; p=0·0010), and 24 months (4·2, 2·0–8·8; p<0·0001). The risk of microbiologically confirmed tuberculosis disease was also higher among children acquiring cytomegalovirus infection before age 3 months (adjusted HR 3·2, 95% CI 1·0–10·6; p=0·048), 6 months (3·9, 1·2–13·0; p=0·027), 12 months (4·4, 1·2–16·3; p=0·027), and 24 months (6·1, 1·3–27·9; p=0·020). In children older than 1 year, the risk of tuberculosis disease was consistently greater in those with high cytomegalovirus loads than in those with low cytomegalovirus loads that were acquired before age 3 months (adjusted HR 2·0 *vs* 3·7; p_trend_=0·0020; both groups compared with cytomegalovirus negative reference) and before age 12 months (2·7 *vs* 3·7; p_trend_=0·0009).

**Interpretation:**

Infants that acquire cytomegalovirus in the first year of life are at high risk of subsequently developing tuberculosis disease. Efforts to prevent tuberculosis in early childhood in high-burden countries might need to deter or delay acquisition of cytomegalovirus perinatally or in the first months of life.

**Funding:**

Bill & Melinda Gates Foundation, MRC South Africa, National Research Foundation South Africa, and Wellcome Trust.

## Introduction

Approximately 1 million children develop tuberculosis disease each year globally, half occurring in early childhood.^1^, ^2^ The risk of tuberculosis disease after recent exposure is greatest before age 5 years,^3^, ^4^, ^5^, ^6^ approaching 20% at 2 years postinfection.^3^ These high progression rates have been ascribed to an immature immune system in early life; however, the mechanisms are not well understood.^7^

The hypothesis that cytomegalovirus acquisition might increase the risk of tuberculosis disease has been posited.^8^ Cytomegalovirus infection is typically asymptomatic in childhood but most people continue to host a latent and chronic infection that might reactivate later in their lifetime.^9^ Cytomegalovirus drives immune activation and dysfunction, increasing lymphocyte-producing and cytokine-producing inflammatory T cells.^9^, ^10^, ^11^ Studies have shown^8^, ^12^, ^13^ that CD4^+^ T-cell activation predicts progression to tuberculosis disease. Additionally, tuberculosis disease and cytomegalovirus have similar age distributions with the greatest risk in infancy and adolescence.^3^, ^4^, ^5^, ^6^, ^9^, ^14^ A case–control study found that children with tuberculosis disease had higher concentrations of cytomegalovirus-specific IFN-γ responses.^15^ Large, population-based, prospective, cohort studies from high-burden settings such as sub-Saharan Africa with serial follow-up testing of cytomegalovirus and tuberculosis disease in infants and young children are not available, but they are needed to further understand the pathogenesis and temporal dynamics between these two hyperendemic pathogens.^8^

**Research in context**

**Evidence before this study**

The risk of tuberculosis disease after recent exposure is greatest in the first few years of life. High tuberculosis progression rates in infancy have been ascribed to an immature immune system; however, the mechanisms responsible for this increased susceptibility are not well understood. Acquisition of viral infections, such as cytomegalovirus, in early life might modulate the immune response. Few studies have investigated acquisition of cytomegalovirus and the subsequent incidence of tuberculosis-related outcomes in young children. A case–control study of 49 infants with tuberculosis disease and 129 matched control infants found that those with tuberculosis disease had a higher cytomegalovirus-specific IFN-γ response, suggesting a possible relationship between these two pathogens. Two retrospective studies among adults in the UK and Uganda found a higher risk of tuberculosis in participants with a positive cytomegalovirus-specific IgG. Whether cytomegalovirus and tuberculosis are epidemiologically related or share common risk factors or exposure events (ie, siblings, household members, and socioeconomic status) remains unknown. Large, prospective, cohort studies from high-burden settings with serial follow-up testing of cytomegalovirus and tuberculosis disease in infants and young children are not available.

**Added value of this study**

To our knowledge, this is the first birth cohort study to investigate cytomegalovirus acquisition and subsequent incident tuberculosis disease. In this community-based, longitudinal study, 963 infants were serially tested for cytomegalovirus (>7000 total tests) and prospectively screened for *Mycobacterium tuberculosis* infection and disease during childhood. More than 40% of infants tested positive for cytomegalovirus by age 1 year and those who were breastfed had a higher risk. Infants with cytomegalovirus infection in the first year of life had a risk of tuberculosis disease after age 1 year that was more than three times higher than the risk in those who had not acquired a cytomegalovirus infection. Children acquiring cytomegalovirus infection early in life were at higher risk of subsequently developing tuberculosis disease throughout childhood, even when restricting the study outcome to microbiologically confirmed tuberculosis disease. A consistent biological gradient was present, showing children with a high cytomegalovirus load were at greatest risk of tuberculosis disease. Tuberculin conversion was not associated with cytomegalovirus infection and it did not mediate tuberculosis disease incidence. This finding suggests that the increased risk of tuberculosis disease in infants with cytomegalovirus infection might be due to immunological alterations from cytomegalovirus rather than a shared exposure of tuberculosis and cytomegalovirus. This study goes beyond previous work to provide a rigorous comprehensive assessment of the interplay between these two pathogens at a population level.

**Implications of all the available evidence**

Our findings suggest that the high tuberculosis disease risk in childhood seen in historical and contemporary studies might be partly due to previous acquisition of cytomegalovirus. Efforts to prevent tuberculosis disease in early childhood in high-burden countries might need to deter or delay acquisition of cytomegalovirus perinatally or in the first months of life.

We aimed to evaluate the acquisition of cytomegalovirus infection in infancy and the development of tuberculosis disease in children. We also aimed to assess whether tuberculin skin test conversion mediated an increased risk of tuberculosis disease due to cytomegalovirus.

## Methods

### Study design and participants

In this prospective, birth cohort study, we enrolled pregnant women who were between 20 and 28 weeks of gestation attending antenatal care in Paarl, a periurban setting outside of Cape Town, South Africa (as previously described).^16^, ^17^ Participants were recruited from two clinics (TC Newman and Mbekweni). Pregnant women who were younger than 18 years and intended to leave the Paarl area within 1 year of starting the study were excluded. Infants were given Bacillus Calmette–Guérin (BCG) vaccination at birth as per national policy. All mothers accessed care in the public sector with a strong primary health-care programme, including mother-to-child HIV prevention and antiretroviral therapy programmes. Follow-up occurred throughout pregnancy and childbirth, and infants were followed up for tuberculosis disease until age 9 years.

All women provided written informed consent at enrolment, which was renewed annually. We obtained ethics approval from the Faculty of Health Sciences Human Research Ethics Committee (University of Cape Town; numbers 401/2009 and 651/2013) and the Provincial Child Health Research Committee.

### Procedures

Comprehensive maternal health questionnaires were administered at enrolment and antenatal (eg, laboratory sampling) data were collected concurrently. Birth information (eg, birthweight, method of delivery, and gestational age) was obtained at delivery. Obstetric care and births took place at the regional hospital in Paarl.

Follow-up visits including clinical examinations were done at age 6, 10, 14 weeks, and 6 and 12 months, and annually thereafter until age 9 years. Data on environmental exposures, household characteristics, respiratory risk factors, anthropometry, and child symptoms were obtained at the scheduled visits. Missed visits were rebooked with a study mobile network system or by study community-based fieldworkers. Mothers were counselled about respiratory symptoms at each visit and advised to attend the study site or contact study staff between scheduled visits whenever the child had a cough or difficulty breathing. Socioeconomic status was comprised of comprehensive composite of asset ownership, household income, employment, and education (appendix 3–4).

All mothers were tested for HIV during pregnancy with an Determine HIV 1/2 rapid HIV antibody test (Abbott Laboratories, Chicago, IL, USA). If positive, a confirmatory ELISA was done. All mothers who were HIV-positive received antiretroviral therapy as per national guidelines and infants of those mothers were tested for HIV infection using PCR (Cobas AmpliPrep system; Roche Molecular Systems, Branchburg, NJ, USA) at age 6 weeks and 6 weeks after the end of breastfeeding. Children were retested at age 18 months with the rapid antibody test.^18^, ^19^

Cytomegalovirus was assessed in children by collecting cytomegalovirus-specific DNA using nasopharyngeal swabs, as described elsewhere.^16^ Nasopharyngeal swabs are less commonly used for detection of congenital cytomegalovirus detection; however, they were found to have a higher yield than dried umbilical cord blood.^20^ Most mothers agreed to intensive follow-up of infants, which involved collection of nasopharyngeal swabs and detection using qPCR done at birth, age 3 and 6 weeks, and age 3, 6, 12, and 24 months. Testing is described further in the appendix (p 5). Laboratory staff were masked to disease status (tuberculosis disease or any other morbidities) of the children during testing. Congenital cytomegalovirus was defined as a positive test at age younger than 3 weeks, with or without symptoms.

Tuberculin skin tests were done at the 6-month visit and then at age 12, 24, 36, 48, and 60 months, and at the time of lower respiratory tract infection.^17^ Tuberculin skin test conversion was defined as an induration reaction greater than or equal to 10 mm, to minimise the risk of misclassification as a result of BCG vaccination or exposure to environmental mycobacteria.^21^ To prevent misinterpretation of boosted skin test reactions as *Mycobacterium tuberculosis* infection due to recurrent tuberculin skin testing, children with a reactive but negative skin test (1–9 mm) were not given another test and were censored for the tuberculin skin test conversion analysis at that point. Children with positive skin tests were screened for tuberculosis disease and referred to local tuberculosis clinics for isoniazid preventive therapy; however, study investigators could not enforce that this was prescribed.

Trained study staff collected sputum smear samples and induced sputum in duplicate for tuberculosis culture and mycobacterial PCR (Xpert MTB/RIF; Cepheid, Sunnyvale, CA, USA) from all children with a tuberculin skin test induration and those who were suspected to have tuberculosis disease. A chest radiograph was taken in all children with suspected pulmonary tuberculosis. Tuberculosis disease was diagnosed by experienced physicians and nurses in local tuberculosis community clinics, and chest radiographs were read and reported by an experienced clinician. We used standardised definitions for diagnostic classification of tuberculosis disease.^22^ The diagnosis of tuberculosis disease required a bacteriological confirmation (positive Xpert, culture, or smear test) for microbiological confirmation. For a clinical diagnosis, a diagnosis must have at least two of the following: chest x-ray consistent with tuberculosis, close tuberculosis exposure or a positive tuberculin skin test, signs and symptoms of tuberculosis, or a positive response to tuberculosis treatment.

### Statistical analysis

Mother–infant pairs were included in the study analyses if the child had at least one cytomegalovirus test result. We summarised continuous variables as medians with IQR and categorical variables using proportions.

For tuberculosis disease incidence, time-to-event data were constructed between birth and the date of tuberculosis. Follow-up was censored at death, development of tuberculosis disease, or end of follow-up (Jan 30, 2021). We compared incidence of tuberculosis disease in groups with and without cytomegalovirus infection at different ages using hazard ratios (HRs) and 95% CIs obtained from Cox proportional hazard models.^23^, ^24^ A two-sample likelihood ratio test was used. We evaluated the proportional hazards assumption by tests of non-zero slope in a generalised linear regression of the scaled Schoenfeld residuals on time. Ties were handled in the Cox models using the Peto–Breslow method.^25^

We used various multivariable models to assess the acquisition of cytomegalovirus and subsequent development of tuberculosis disease after adjustment for the effects of confounding variables. The primary outcome was tuberculosis disease incidence after age 1 year or after age 6 months in children with and without cytomegalovirus infection and, in our models, we varied the timing of cytomegalovirus acquisition on the basis of the child's age at the time of the test (age 3 and 6 weeks, and age 3, 6, 12, and 24 months). Cytomegalovirus positivity is cumulative and therefore these cutoffs include all positive tests before that age (eg, 3 months indicates all positive tests at and before that timepoint). For our secondary analyses, we varied the follow-up period for tuberculosis based on the time from birth (follow-up from age 6 months to 1 year, 1–2 years, 2–9 years, and at any timepoint). We estimated the population attributable proportion of tuberculosis disease after age 1 year due to cytomegalovirus in infancy using standard formulas (appendix pp 5, 18).^26^

To assess whether there was a dose-response between cytomegalovirus load values at a specific timepoint and risk of tuberculosis disease, we calculated the mean of all cytomegalovirus load values from each participant at and before that timepoint. We then took the log of each individual mean viral-load value and split participants into low and high log viral load values by calculating the median of all positive values. We then compared tuberculosis disease risk among cytomegalovirus-negative participants with those with low and high log viral-load values. We varied the follow-up period for tuberculosis disease on the basis of time from birth.

Lastly, to investigate whether cytomegalovirus and tuberculosis disease have shared exposures, we assessed the association between cytomegalovirus positivity and tuberculin conversion at age younger than 1 year using logistic regression models. We built multivariable models to include all relevant variables related to tuberculin conversion in this cohort.^17^ We compared tuberculin skin test indurations among children with and without cytomegalovirus infection at different age timepoints using Mann–Whitney *U* tests. All analyses were performed using Stata (version 14.1).

### Role of the funding source

The funders of the study had no role in study design, data collection, data analysis, data interpretation, or writing of the report.

## Results

**Figure 1:**
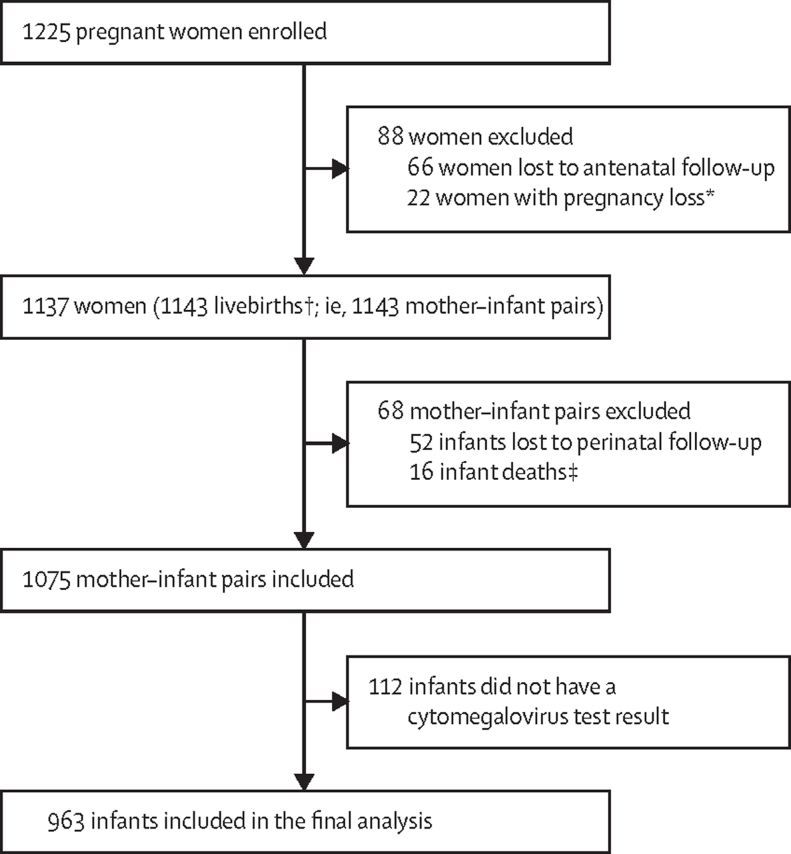
Trial profile *Loss of pregnancy due to miscarriage, stillbirth, or intrauterine death (23 infants including one set of twins). †Including four pairs of twins and one set of triplets. ‡No postnatal data collected.

Between March 5, 2012, and March 31, 2015, 1225 pregnant women were recruited and enrolled in the birth cohort (figure 1). 88 (7%) women were excluded because of loss to antenatal follow-up or pregnancy losses. Of 1143 livebirths, 68 (6%) mother–infant pairs were excluded because of loss to perinatal follow-up (n=52) and infant death (n=16), and 112 infants (15%) were enrolled but did not have a valid cytomegalovirus test. In total, 963 children (including two living with HIV and 224 exposed to HIV and uninfected) were serially tested for cytomegalovirus and included in the final study analyses (table 1). During follow-up, 7186 cytomegalovirus measurements were taken (median six tests per child, IQR 2–11). Most (6224 [87%]) tests were performed before age 1 year. Characteristics of excluded and included infants were similar; however, excluded participants were more likely to be from a higher income family and less likely to be breastfed (appendix pp 6–7).

**Table 1:** Sociodemographic and clinical characteristics of 963 mother–infant pairs, stratified by community site of enrolment

			**TC Newman clinic (n=382)**	**Mbekweni clinic (n=581)**	**All participants (n=963)**
**Infant characteristics**					
Sex					
	Boys		213 (56%)	293 (50%)	506 (53%)
	Girls		169 (44%)	288 (50%)	457 (48%)
Breastfed at any time			379 (99%)	508 (87%)	887 (92%)
Median gestational age, weeks			39 (37 to 40)	39 (38 to 40)	39 (38 to 40)
	Preterm birth, <37 weeks		61 (16%)	94 (16%)	155 (16%)
Living with HIV			0	2 (<1%)	2 (<1%)
Birthweight					
	Low birthweight, <2·5 kg		71 (19%)	68 (12%)	139 (14·4%)
	Median birthweight, kg		3·0 (2·6 to 3·3)	3·2 (2·8 to 3·5)	3·1 (2·7 to 3·4)
Infant size					
	Median length		50·0 (47·0 to 52·0)	50·0 (48·0 to 52·0)	50·0 (48·0 to 52·0)
	Median weight-for-age Z score*		−0·73 (−1·40 to −0·07)	−0·43 (−1·20 to 0·19)	−0·54 (−1·29 to 0·07)
	Underweight, Z score <–2		41 (11%)	45 (8%)	86 (9%)
	Healthy weight, Z score −2 to 2		338 (88%)	520 (90%)	858 (89%)
	Overweight, Z score >2		2 (<1%)	13 (2%)	15 (2%)
	Missing Z score		0	3 (<1%)	3 (<1%)
**Maternal characteristics**					
Median age, years			24·7 (21·2 to 29·3)	26·9 (22·6 to 31·7)	25·9 (22·1 to 30·9)
Married or cohabitating			167 (44%)	219 (38%)	386 (40%)
Living with HIV			12 (3%)	214 (37%)	226 (23%)
Median CD4 count			567 (370 to 679)	406 (283 to 591)	411 (285 to 594)
WHO stages of HIV infection					
	1		7 (41%)	83 (57%)	90 (58%)
	2		2 (12%)	27 (19%)	29 (19%)
	3		1 (6%)	30 (21%)	31 (20%)
	4		7 (41%)	5 (3%)	5 (3%)
Tuberculosis treatment during pregnancy			19 (5%)	23 (4%)	42 (4%)
Ever diagnosed with tuberculosis			12 (3%)	24 (4%)	36 (4%)
Smoking during pregnancy			178 (47%)	25 (4%)	203 (21%)
Education					
	Finished only primary school		31 (8%)	44 (8%)	75 (8%)
	Did not finish secondary school		209 (55%)	320 (55%)	529 (55%)
	Finished secondary school		142 (37%)	218 (38%)	360 (37%)
Employed			101 (26%)	139 (24%)	240 (25%)
**Household characteristics**					
	Socioeconomic status†				
		Low	70 (18%)	175 (30%)	245 (25%)
		Moderate-low	103 (27%)	150 (26%)	253 (26%)
		Moderate-high	100 (26%)	146 (25%)	246 (26%)
		High	101 (26%)	109 (19%)	210 (22%)
		Missing	8 (2%)	2 (<1%)	10 (1%)
	Household income, South African Rand per month				
		<1000	127 (33%)	253 (44%)	380 (40%)
		1000–5000	199 (52%)	274 (47%)	472 (49%)
		>5000	56 (15%)	55 (10%)	111 (12%)
	Housing				
		Shack or informal dwelling	118 (31%)	246 (42%)	363 (38%)
		House or flat	264 (69%)	336 (58%)	600 (62%)
	Crowding, people per household				
		Median people per household	3 (2 to 4)	2 (2 to 4)	3 (2 to 4)
		≤3	89 (23%)	245 (42%)	334 (35%)
		4–5	144 (38%)	170 (29%)	314 (33%)
		>5	147 (38%)	167 (29%)	314 (33%)
		Missing	1 (<1%)	0	1 (<1%)

Data are n (%) and median (IQR). Percentages might not equal 100% because within-column percentages were rounded to the nearest integer. Column totals vary across different characteristics because of missing values for some participants.

* We derived Z scores from WHO child growth standards at birth and at every follow-up visit; we used the median of all the weight-for-age Z scores for each child to summarise nutrition status over the duration of follow-up.

† Socioeconomic status included a comprehensive composite of asset ownership, household income, employment, and education. Derivation of the socioeconomic status classifications is shown in the appendix (pp 3–4).

The prevalence of congenital cytomegalovirus at age younger than 3 weeks was 2% (18 of 816). Cytomegalovirus positivity increased continuously with age from 3% (27 of 825) by age 6 weeks to 21% (183 of 882) by 3 months, 35% (315 of 909) by 6 months, and 42% (390 of 933) by 12 months. Breastfeeding was strongly related to cytomegalovirus positivity by 12 weeks (22% for breastfed *vs* 8% for non-breastfed children; p=0·0040; appendix p 22), 6 months (37% *vs* 14%; p<0·0001), and 12 months (44% *vs* 14%; p<0·0001). Children who were breastfed for less than 1 month were more likely to be cytomegalovirus-positive compared with non-breastfed children (adjusted odds ratio [OR] 4·4, 95% CI 2·0–9·1; p<0·0001). This finding was greater for children who were breastfed for 1 month or longer compared with non-breastfed children (6·8, 3·3–14·2; p<0·0001). Cytomegalovirus infection by age 1 year was not associated with socioeconomic status (adjusted OR 1·0, 95% CI 0·9–1·2; p=0·24), the number of children in the household younger than 5 years (1·1, 0·9–1·3; p=0·60) or aged 5–18 years (1·0, 0·9–1·1; p=0·89), or family size (1·0, 0·9–1·1; p=0·12; appendix pp 8–9). Maternal HIV was also not associated with cytomegalovirus infection in children before age 1 year (adjusted OR, 1·1 95% CI 0·8–1·6; p=0·71).

Mother–infant pairs were followed up for a median of 6·9 years (IQR 6·0–7·8). Over 6270 child-years of follow-up, 75 [8%] of 963 children were diagnosed with tuberculosis disease (1196 children per 100 000 person-years, 95% CI 954–1500). One child had disseminated tuberculosis disease and the remaining 74 children had pulmonary tuberculosis disease. 17 (23%) of 75 had microbiologically confirmed tuberculosis disease. The median age at diagnosis of tuberculosis disease was 1·0 year (IQR 0·5–2·4) and 43 children developed tuberculosis disease before the age of 1 year. We found no difference in tuberculosis disease incidence in children exposed to HIV versus unexposed children (adjusted HR 1·0, 95% CI 0·5–2·1; p=0·51). 29 (28%) of 103 children with tuberculin skin test conversion were recorded as given or adherent to preventive therapy following referral to clinical tuberculosis services.

**Figure 2:**
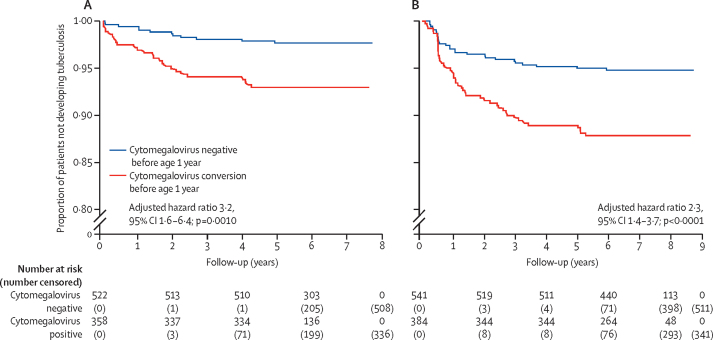
Acquisition of cytomegalovirus and the development of tuberculosis disease Adjusted for child sex, maternal HIV status, and study site. (A) Follow-up time after age 1 year for development of tuberculosis disease (until age 9 years). (B) Follow-up was not restricted and included all follow-up for children aged 0–9 years.

In our primary analyses of children after age 6 months, those with a positive cytomegalovirus test were at a consistently higher risk of developing tuberculosis disease than those with a negative cytomegalovirus test, regardless of timing of cytomegalovirus acquisition, except by age 3 weeks and younger (table 2). When considering follow-up for tuberculosis disease in children after age 1 year in a multivariable model adjusting for maternal HIV, sex, and study site, the risk of tuberculosis disease was higher in those with cytomegalovirus infection by age 6 weeks (adjusted HR 4·1, 1·2–13·8; p=0·022), 3 months (2·8, 1·4–5·8; p=0·0040), 6 months (3·6, 1·7–7·3; p<0·0001), 12 months (3·2, 1·6–6·4; p=0·0010), and 24 months (4·2, 2·0–8·8; p<0·0001; figure 2).

**Table 2:** Adjusted hazard ratios of tuberculosis disease in early childhood by the timing of cytomegalovirus acquisition

		Cytomegalovirus acquisition by age 3 weeks and younger*	**Cytomegalovirus acquisition by age 6 weeks and younger**	**Cytomegalovirus acquisition by age 3 months and younger**	**Cytomegalovirus acquisition by age 6 months and younger**	**Cytomegalovirus acquisition by age 12 months and younger**	**Cytomegalovirus acquisition by age 24 months and younger**
**Primary analyses: follow-up for tuberculosis disease**							
After age 6 months		3·7 (1·1–11·9)	3·3 (1·2–9·3)	2·3 (1·3–4·1)	3·1 (1·8–5·5)	2·7 (1·6–4·6)	3·6 (2·0–6·4)
	Tuberculosis episodes	43	44	50	52	57	58
	Follow-up, person–years	5334	5382	5734	5904	6037	6197
After age 1 year		3·9 (0·9–16·4)	4·1 (1·2–13·8)	2·8 (1·4–5·8)	3·6 (1·7–7·3)	3·2 (1·6–6·4)	4·2 (2·0–8·8)
	Tuberculosis episodes	27	27	31	33	37	38
	Follow-up, person–years	4544	4584	4882	5028	5138	5276
**Secondary analyses: follow-up for tuberculosis disease**							
Age 6 months to 1 year		3·2 (0·4–24·6)	2·0 (0·3–15·3)	1·6 (0·6–4·2)	2·5 (1·0–6·2)	2·0 (0·8–4·9)	2·6 (1·0–6·8)
	Tuberculosis episodes	16	17	19	19	20	20
	Follow-up, person–years	791	797	851	877	898	921
Age 1–2 years		6·8 (0·8–56·8)	10·9 (2·1–55·3)	3·8 (1·1–13·2)	4·0 (1·2–13·4)	5·6 (1·6–20·2)	8·4 (1·9–37·4)
	Tuberculosis episodes	8	8	10	12	14	15
	Follow-up, person–years	1548	1560	1664	1713	1753	1799
Age 2–9 years		2·7 (0·4–20·5)	1·8 (0·2–13·9)	2·5 (1·0–6·0)	3·3 (1·4–8·1)	2·4 (1·0–5·5)	3·0 (1·2–7·2)
	Tuberculosis episodes	19	19	21	21	23	23
	Follow-up, person–years	3776	3811	4058	4179	4270	4386
At any timepoint		2·8 (0·9–9·1)	3·1 (1·2–7·8)	2·1 (1·3–3·4)	2·3 (1·4–3·7)	2·3 (1·4–3·7)	2·9 (1·7–4·7)
	Tuberculosis episodes	55	58	66	69	74	75
	Follow-up, person–years	5339	5386	5739	5910	6042	6203

Data are adjusted hazard ratio (95% CI) or n. All models are adjusted for child sex, study site, and maternal HIV. The reference values for each row are children that are cytomegalovirus negative at that timepoint. Cox regression models were performed with differing timing of cytomegalovirus positivity and follow-up time for tuberculosis disease. Sensitivity analysis adjusting for further risk factors (including socioeconomic status, birthweight, household exposure, tuberculin conversion, and number of cytomegalovirus tests) can be seen in the appendix (pp 12–13). Excluding congenital cytomegalovirus infections did not affect these results and can also be seen in the appendix (pp 14–15). Cytomegalovirus positivity is cumulative and therefore, these cutoffs include all positive tests before that age.

* Defined as congenital cytomegalovirus.

These associations did not change significantly after adjustment for socioeconomic status, birthweight, household tuberculosis exposure, preventive therapy, number of cytomegalovirus tests, tuberculin conversion, pneumonia, and the exclusion of congenital cytomegalovirus infections (appendix pp 14–15).

When restricting our analysis to other follow-up time periods, we found similar results. Children who acquired cytomegalovirus were at high risk of subsequent tuberculosis disease at most timepoints for cytomegalovirus acquisition and most restrictions of follow-up to tuberculosis disease (table 2).

We estimated that 48% (95% CI 20·5–69·3) of tuberculosis disease in children older than 1 year in our birth cohort were attributable to a cytomegalovirus infection before age 1 year.

When restricting our outcome to microbiologically confirmed tuberculosis (including all follow-up), the risk of tuberculosis disease was higher among children acquiring cytomegalovirus infection before age 3 months (adjusted HR 3·2, 95% CI 1·0–10·6; p=0·048), 6 months (3·9, 1·2–13·0; p=0·027), 12 months (4·4, 1·2–16·3; p=0·027), and 24 months (6·1, 1·3–27·9; p=0·020).

**Figure 3:**
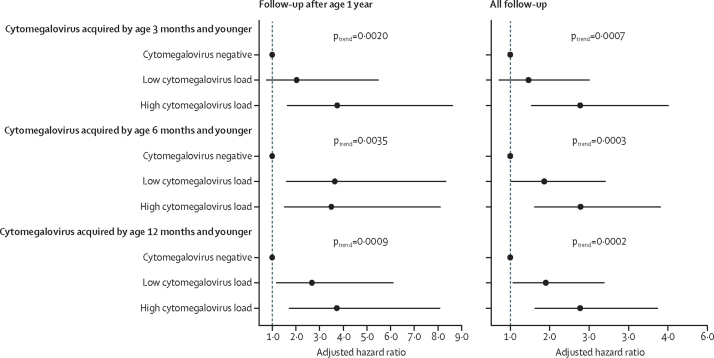
Dose-response between timing of cytomegalovirus positivity, cytomegalovirus load, and the risk of developing tuberculosis disease during childhood We did not include congenital cytomegalovirus (positive test by age 3 weeks and younger) or cytomegalovirus by age 6 weeks and younger because of low statistical power. Follow-up time was restricted by timing from birth. All models were adjusted for child sex, study site, and maternal HIV. All number of events, follow-up time, and effect estimates can be found in the appendix (pp 10–11). The higher and lower values with quantitative thresholds were different for each timepoint (appendix p 21).

There was a dose-response relationship between cytomegalovirus load and risk of tuberculosis disease at most but not all timepoints for cytomegalovirus acquisition (figure 3; appendix pp 10–11). When restricting follow-up to children older than 1 year, the risk of tuberculosis disease was consistently greater in those with high cytomegalovirus loads than in those with low cytomegalovirus loads that were acquired by age 3 months and younger (adjusted HR 3·7 *vs* 2·0**;** p_trend_=0·0020) and 12 months and younger (3·7 *vs* 2·7; p_trend_=0·0009). When including all follow-up timepoints, the risk of tuberculosis disease was higher in infants with high cytomegalovirus load than in those with cytomegalovirus-negative tests, regardless of the timing of cytomegalovirus infection.

We did not find an association between cytomegalovirus positivity in children aged younger than 6 weeks and tuberculin conversion (adjusted OR 1·6, 95% CI 0·5–5·1; p=0·39). There was also no association among children with and without a positive cytomegalovirus test aged younger than 3 months (1·3, 0·8–2·2; p=0·27), 6 months (1·4, 0·9–2·2; p=0·14), and 1 year (1·2, 0·8–1·9; p=0·33). Tuberculin skin test indurations did not differ among children with and without cytomegalovirus aged younger than 3 weeks (p=0·31), 6 weeks (p=0·82), 3 months (p=0·51), 6 months (p=0·62), and 12 months (p=0·95; appendix p 28).

Multivariable models assessing cytomegalovirus acquisiton before age 1 year that included tuberculin conversion (adjusted HR 3·7, 95% CI 1·7–8·4) or household tuberculosis exposure (3·2, 1·6–6·5) did not appreciably alter the risk of tuberculosis disease compared with models not adjusting for tuberculin conversion (3·2, 1·6–6·4). This finding suggests that tuberculin conversion and household tuberculosis exposure did not mediate the association seen between cytomegalovirus acquisition and subsequent tuberculosis disease.

## Discussion

In this prospective, population-based, birth cohort study in South Africa, 42% of infants were cytomegalovirus-positive in the first year of life and had a higher risk of subsequent tuberculosis disease throughout childhood. We found this association even when we restricted the outcome to microbiologically confirmed tuberculosis disease. Children with high cytomegalovirus load were at especially high-risk, implying a biological gradient. This finding was not modified by tuberculin conversion or known tuberculosis exposure, suggesting that immune dysfunction—not a common exposure—probably explains these results. Prevention of cytomegalovirus acquisition early in life might be an important immunomodulatory intervention to protect young children from subsequently developing tuberculosis disease.

Cytomegalovirus prevalence in impoverished communities can reach more than 50% in the first 1–2 years of life.^9^, ^14^, ^27^, ^28^ Our findings suggest that breastfeeding is probably a major transmission pathway for acquisition of cytomegalovirus in South Africa. Additionally, approximately 2% of participants were diagnosed with congenital cytomegalovirus. However, there was no evidence of other methods of transmission that have been postulated, such as transmission from siblings or family members, other than the mother.^14^, ^29^, ^30^ Furthermore, socioeconomic status, household income, and family size were not associated with cytomegalovirus acquisition, indicating that several factors commonly related to tuberculosis disease were not associated with cytomegalovirus acquisition.

Cytomegalovirus is often asymptomatic and adversely affects the immune system through cytomegalovirus-specific memory CD4^+^ and CD8^+^ T-cell activation.^7^, ^8^, ^29^, ^31^, ^32^ Studies^8^, ^15^ have postulated that children with cytomegalovirus infection might have an increased risk of tuberculosis disease; however, there are few epidemiological studies supporting this hypothesis. A case–control study^15^ of 49 infants who developed tuberculosis disease and 129 healthy infants found that those developing tuberculosis disease had higher cytomegalovirus-specific IFN-γ responses. Two retrospective studies^33^, ^34^ among adults in the UK and Uganda found a higher risk of tuberculosis disease in participants with a positive cytomegalovirus-specific IgG. The timing of cytomegalovirus acquisition (whether in the past few years or through reactivation of a previous exposure) in these studies is unclear. Our results provide further evidence of the relationship between cytomegalovirus and tuberculosis disease, namely that this association might be highly relevant to tuberculosis pathogenesis and that some viral infections, such as cytomegalovirus, are important to study further.^35^

Shared exposure to tuberculosis disease and cytomegalovirus is unlikely to explain the higher childhood tuberculosis risk we observed among participants with cytomegalovirus during infancy. Cytomegalovirus was not associated with tuberculin conversion in infancy. Furthermore, tuberculin conversion and household tuberculosis exposure did not act as a mediator between the acquisition of cytomegalovirus and progression to tuberculosis. If children acquired cytomegalovirus from the same source of tuberculosis exposure (eg, at daycare or from a household visitor), this finding would be most apparent in tuberculin conversion results. The increased risk of tuberculosis disease, but not recent *M tuberculosis* infection or exposure, suggests that immunological alterations due to cytomegalovirus infection might increase the risk of progression from *M tuberculosis* infection to disease in young children. Alternatively, an early immunological deficit in utero or in the first few days of life might predispose to infection or disease with each pathogen. Further studies are needed to explore underlying mechanisms for this association.

We found consistent evidence suggesting that a dose-response was present between cytomegalovirus load values and tuberculosis risk in early childhood for different timepoints and follow-up time periods. Such a biological gradient should be unaffected by diagnostic biases because children tested for cytomegalovirus were not selected on the basis of a tuberculosis diagnosis. A case–control study of 25 patients with tuberculosis and 256 controls without tuberculosis in urban Uganda found that the odds of tuberculosis disease increased with levels of cytomegalovirus-specific humoral responses.^34^ Our results, in combination with these findings among adults, suggests that the cytomegalovirus exposure load might modify subsequent risk of tuberculosis disease.

The strengths of our study include the prospective design (thereby eliminating potential recall biases) and the large community-based sample. Longitudinal measurement of *M tuberculosis* infection and disease and cytomegalovirus are unique, to our knowledge, allowing us to understand the temporal occurrence of these diseases and to investigate pathways of both diseases. Intense surveillance of tuberculosis disease, including the availability of tuberculin skin tests, chest radiographs, smear and culture, and Xpert MTB/RIF, and detection of a range of other diseases would to lead to high tuberculosis detection in this difficult to diagnose group.

Our study also has limitations. First, we did not assess maternal cytomegalovirus status before and during pregnancy, which is a potential modifier of infant cytomegalovirus status. Second, boosting with BCG vaccination or repeated skin tests might lead to false-positive conversion results and, to address this issue, any child with a positive skin test reaction did not have a repeat skin test. Additionally, we used a conservative conversion cutoff (10 mm induration).^36^ If present, BCG boosting is unlikely to be differentially seen in children with and without cytomegalovirus conversion. Third, 10% of our cohort were not tested for cytomegalovirus and were excluded. Participants who were excluded were similar to those in the study population; however, selection bias is possible. Lastly, although studies suggest reasonable yield of nasopharyngeal PCR for congenital cytomegalovirus detection,^20^ reduced sensitivity is a possibility, which could bias the association between cytomegalovirus and tuberculosis disease towards the null.

In conclusion, over 40% of our cohort were cytomegalovirus-positive in the first year of life, and our findings suggest children who have acquired cytomegalovirus are at increased risk of subsequently developing tuberculosis disease. Children with an elevated cytomegalovirus load might be at particularly high risk of tuberculosis disease. Efforts to prevent tuberculosis disease in early childhood in high-burden countries might need to deter or delay acquisition of cytomegalovirus perinatally or in the first months of life.

## Data sharing

The data used for this analysis can be made available upon reasonable request once all relevant substudies from the Drakenstein Child Health Study are reported and completed. The data dictionary can be made available upon request to the corresponding author.

## Declaration of interests

HJZ reports grants from the Bill & Melinda Gates Foundation, the South African Medical Research Council, the South African National Research Foundation, and the National Institutes of Health. All other authors declare no competing interests.
