# Correction to *Lancet Glob Health* 2021; 9: e1740–49

**DOI:** 10.1016/S2214-109X(21)00541-6

**Published:** 2021-11-17

*Martinez L, Nicol MP, Wedderburn CJ, et al. Cytomegalovirus acquisition in infancy and the risk of tuberculosis disease in childhood: a longitudinal birth cohort study in Cape Town, South Africa.* Lancet Glob Health *2021;* 9: *e1740–49*—In this Article, the follow-up in table 2 should have been 4935, 4979, 5304, 5461, 5590, and 5732 person–years after age 6 months; 392, 395, 421, 434, 445, and 456 person–years at age 6 months to 1 year; and 767, 773, 825, 849, 869, and 891 person–years at age 1–2 years. This correction has been made as of Nov 17, 2021.

