# Supplementary material for: Cytomegalovirus acquisition in infancy and the risk of tuberculosis disease in childhood: a longitudinal birth cohort study in Cape Town, South Africa
Source: Lancet Glob Health. 2021 Nov 16;9(12):e1740–9. doi: 10.1016/S2214-109X(21)00407-1 (PMC8609281; doi:10.1016/S2214-109X(21)00407-1)
Supplement: Supplementary appendix [file mmc1.pdf]

THE LANCET
Global Health

Supplementary appendix

This appendix formed part of the original submission and has been peer reviewed. We post it as supplied by the authors.

Supplement to: Martinez L, Nicol MP, Wedderburn CJ, et al. Cytomegalovirus acquisition in infancy and the risk of tuberculosis disease in childhood: a longitudinal birth cohort study in Cape Town, South Africa. *Lancet Glob Health* 2021; **9:** e1740–49.

Supplementary Appendix

Supplement to: Cytomegalovirus Acquisition in Infancy and the Risk of Tuberculosis in Childhood: A Longitudinal Birth Cohort in Cape Town, South Africa

Table of Contents.

1. Further Methodological Information.

2. Table S1. Comparison of excluded and included participants into the study.

3. Table S2. Multivariable logistic regression assessing the relationship between cytomegalovirus positivity at different child ages and secondary characteristics.

4. Table S3. Dose-response relationship between timing of cytomegalovirus positivity, level of cytomegalovirus load, and the risk of developing tuberculosis during childhood

5. Table S4. Sensitivity analyses showing adjusted hazard ratios with adjustment of tuberculin conversion through a censoring approach (main analysis) and not using a censoring approach (sensitivity analysis), by timing of cytomegalovirus positivity.

6. Table S5. Sensitivity analyses showing adjusted hazard ratios with additional adjustment and exclusion of congenital cytomegalovirus infections.

7. Table S6. Multivariable mediation analyses of tuberculin conversion and household tuberculosis exposure on the relationship between cytomegalovirus and risk of tuberculosis development.

8. Table S7. Cytomegalovirus positivity in the intensive and non-intensive cohort.

9. Table S8. Population attributable fractions of tuberculosis disease at distinct time points due to cytomegalovirus at corresponding time points.

10. Table S9. P-value tests of nonzero slope in a generalized linear regression of the scaled Schoenfeld residuals on time

11. Table S10. Median cytomegalovirus load and categorization cutoffs of low versus high viral loads

12. Table S11. Timing of cytomegalovirus positivity and breastfeeding.

13. Table S12. Children diagnosed with tuberculosis and CMV positivity at a given time point.

14. Table S13. Cytomegalovirus status based on the timing of cytomegalovirus testing and the study site.

15. Table S14. STROBE Statement.

16. Table S15. Number of censored participants in the primary and sensitivity analyses.

17. Table S16. Tuberculin skin test indurations among children acquiring and not acquiring cytomegalovirus.

1. Further Methodological Information.

Classification of Socioeconomic Status:

These classifications have been described in detail previously in Koen et al, 2014 entitled "Intimate partner violence: associations with low infant birthweight in a South African birth cohort". We describe it briefly below and have added text to the Supplementary Appendix also describing these definitions.

Briefly, a survey was adapted from the South African Stress and Health Study (SASH) (Myer et al. 2008).

This survey assessed education and income; access to governmental financial assistance; household composition; and available amenities (including electricity, running water, electric stove and a functional telephone).

A composite score of socioeconomic status was developed. Four sociodemographic variables (as assessed by the SES questionnaire) were used to generate this score, described below.

1) For the assessment of educational attainment, participants were given scores as follows:
Primary education: 0
Some secondary education: 1
Completed secondary education: 2
Any tertiary education: 3

2) For the assessment of employment status participants were given scores as follows:
Currently unemployed: 0
Currently employed: 1

3) For the assessment of household income participants were given scores as follows:
Total household income of less than 1,000 South African rand per month: 0
Monthly income between 1,000 and 5,000 South African rand: 1
Households with an income > 5,000 South African rand per month: 3.

4) For the assessment of assets and market access participants were given scores as follows:

A composite asset index was calculated as the sum of assets/infrastructure and market access. In order to assess assets/infrastructure, participants were requested to indicate the availability of the following household resources and amenities (a score of 1 was assigned for each available item): electricity, a tap or running water, a domestic servant, a flush toilet inside, a built-in kitchen sink, an electric stove or hotplate, a working telephone (including cellphone), at least 1 motor car or

truck, a motorcycle or scooter, and/or a bicycle. The composite score for assets/infrastructure was then obtained by summing the individual item scores.

The total market access score was assessed by these items: shopping at supermarkets, using any financial services (such as bank account, ATM card or credit card) and/or having an account at a retail store. Each affirmative item was assigned a score of 1, and the total market access score derived by adding these individual scores.

The grand total SES score was then generated as follows:

Standardized income + standardized education + standardized assets + (0.5*employment) = Composite SES Score

References.

Koen N, Wyatt GE, Williams JK, Zhang M, Myer L, Zar HJ, Stein DJ. Intimate partner violence: associations with low infant birthweight in a South African birth cohort. Metabolic brain disease. 2014 Jun; 29 (2): 281-99.

Myer L, Stein DJ, Grimsrud A, Seedat S, Williams DR. Social determinants of psychological distress in a nationally-representative sample of South African adults. Social science & medicine. 2008 Apr 1; 66 (8): 1828-40.

<u>Tuberculosis diagnosis:</u>

Children were followed up for tuberculosis disease from birth. Trained study staff collected sputum smear specimens and induced sputum in duplicate for tuberculosis culture and mycobacterial PCR investigation (Xpert MTB/RIF; Cepheid, Sunnyvale, CA, USA) from all children with a tuberculin skin test induration of at least 10 mm (conducted at 6-month intervals in the first year of life and annual visits until five years of age, subsequently), and from children who were suspected to have tuberculosis disease. A chest radiograph was taken in all children with suspected pulmonary tuberculosis.

Continuous surveillance for tuberculosis was implemented at local clinics and at Paarl hospital. Tuberculosis disease was diagnosed by experienced physicians and nurses in local tuberculosis community clinics, and chest radiographs were read and reported by an experienced clinician. We crosschecked tuberculosis diagnoses identified as part of the study with notification databases by the local and national ministries of health for reporting comprehensiveness.

<u>Dose-response analysis:</u>

For the dose-response analysis, we attempted to categorize participants with either 'Low' or 'High' viral load. To do this, we calculated the mean of all viral load values among participants with multiple viral loads. We used this mean value for these participants for this specific analysis. We then calculated the log of these mean values for all participants. Then, to categorize participants into 'Low' and 'High' log viral load values, we calculated the median of all positive cytomegalovirus values (children testing negative at that time point were excluded during this calculation). These median values can be seen in the above table.

Cytomegalovirus testing:

Cytomegalovirus was assessed in children through cytomegalovirus-specific DNA using nasopharyngeal swabs as described previously.22 Nasopharyngeal swabs are less commonly used for detection of congenital cytomegalovirus detection however have been found to have higher yield compared to dried umbilical cord blood in previous studies. In a subgroup of participants, whose mother agreed to intensive follow-up, nasopharyngeal swabs were collected every two weeks for the first year of life. All children had nasopharyngeal swabs taken at birth and every 6 months. Laboratory staff were masked to the disease status (tuberculosis disease or any other morbidities) of children during testing. Congenital cytomegalovirus was defined as a positive test prior to the first 3 weeks of life, with or without symptoms.

Population Attributable Fraction:

We estimated the population attributable fraction of tuberculosis disease after six months of age due to cytomegalovirus in infancy using standard formulas (Rockhill, 1998). The prevalence of exposure is represented as cytomegalovirus infection in the first 1 year of life; the relative risk is represented as the adjusted hazard ratio between cytomegalovirus in infancy (<1 year of age) and tuberculosis disease after 1 year of age. We calculated additional population attributable fractions for other timepoints which are presented below.

Rockhill B, Newman B, Weinberg C. Use and misuse of population attributable fractions. American Journal of Public Health. 1998 Jan; 88 (1): 15-9.

2. Table S1. Comparison of excluded and included participants into the study.

Variable	Included participants (N=963)	Excluded participants (N=180)	All Participants (N=1,143)
	[n (%)]	[n (%)]	[n (%)]
**Infant characteristics**			
Male	505 (52.5)	83 (46.6)	588 (51.6)
Low birthweight, <2.5 kilograms	135 (14.1)	35 (19.7)	172 (15.1)
Preterm birth, <37 weeks	148 (16.3)	42 (18.1)	190 (16.7)
HIV-positive	2 (0.2)	0 (0)	2 (0.2)
Breastfed	831 (92.1)	161 (90.5)	1,047 (91.8)
Median weight-for-age Z-score (IQR)	-0.54 (-1.29, 0.07)	-0.57 (-1.31, 0.02)	-0.55 (-1.31, 0.07)
**Maternal characteristics**			
Age, years (continuous)	25.9 (22.1, 30.9)	24.5 (21.7, 29.7)	25.8 (22.0, 30.8)
Married or cohabitating	360 (39.7)	100 (42.6)	460 (40.3)
HIV-positive	218 (24.0)	31 (13.2)	249 (21.8)
Tuberculosis treatment during pregnancy	41 (4.5)	7 (3.0)	48 (4.2)
Ever diagnosed with tuberculosis	35 (3.9)	6 (2.6)	41 (3.6)
Maternal smoking during pregnancy	189 (21.0)	73 (31.3)	262 (23.1)
**Household Characteristics**			
Household income, rand per month			

<1000	360 (39.7)	70 (29.8)	430 (37.7)
1000–5000	440 (48.5)	119 (50.6)	559 (49.0)
>5000	107 (11.8)	46 (19.6)	153 (13.4)
Formal housing			
Shack or hokkie	340 (37.5)	83 (35.3)	235 (20.6)
House or flat	567 (62.5)	152 (64.7)	907 (79.4)
Crowding, persons per household			
3 or fewer	310 (34.2)	73 (31.1)	383 (33.5)
4 or 5	302 (33.3)	83 (35.3)	385 (33.7)
More than 5	294 (32.4)	78 (33.2)	372 (32.6)
Missing	1 (0.1)	1 (0.4)	2 (0.2)

3. Table S2. Multivariable logistic regression assessing the relationship between cytomegalovirus positivity at different child ages and secondary characteristics

Variable	3 weeks and before	6 weeks and before	3 months and before	6 months and before	12 months and before
Socioeconomic status summation score‡*	0.98 (0.76–1.26)	1.01 (0.88–1.17)	1.00 (0.92–1.08)	1.05 (0.98–1.12)	1.06 (0.99–1.13)
Socioeconomic status‡					
Lowest	1 (Referent)	1 (Referent)	1 (Referent)	1 (Referent)	1 (Referent)
Moderate low	0.97 (0.25–3.65)	1.72 (0.75–3.95)	1.33 (0.84–2.12)	1.11 (0.75 -1.64)	1.16 (0.80–1.68)
Moderate high	0.76 (0.18–3.27)	1.49 (0.63–3.52)	1.08 (0.67–1.75)	0.95 (0.64–1.41)	1.00 (0.69–1.46)
Highest	0.67 (0.13–3.58)	1.12 (0.43–2.95)	1.05 (0.63–1.75)	1.28 (0.85–1.94)	1.43 (0.96–2.12)
Missing	…	…	0.41 (0.05–3.37)	0.82 (0.20–3.30)	0.99 (0.27–3.65)
Household income, rand per month					

<1000	1 (Referent)	1 (Referent)	1 (Referent)	1 (Referent)	1 (Referent)
1000–5000	1..35 (0.46–3.95)	0.85 (0.44–1.62)	1.06 (0.74–1.52)	1.07 (0.79–1.44)	1.13 (0.85–1.51)
>5000	…	0.93 (0.36–2.40)	0.89 (0.51–1.56)	1.16 (0.74–1.84)	1.20 (0.77–1.87)
Crowding, persons per household*	1.04 (0.86–1.26)	1.08 (0.98–1.19)	1.03 (0.97–1.10)	1.03 (0.98–1.08)	1.05 (0.99–1.10)
Children <5 years per household	1.38 (0.75–2.52)	1.16 (0.80–1.69)	1.11 (0.90–1.38)	1.02 (0.85–1.24)	1.07 (0.89–1.28)
Children 5 years or older per household	0.81 (0.48–1.35)	1.02 (0.80–1.30)	1.02 (0.89–1.17)	1.02 (0.91–1.14)	1.01 (0.91–1.13)

‡ Socioeconomic status comprised a comprehensive composite of asset ownership, household income, employment, and education.

* This was included in the multivariable model as a continuous variable

4. Table S3. Dose-response relationship between timing of cytomegalovirus positivity, level of cytomegalovirus load, and the risk of developing tuberculosis during childhood†.

	Timing of Cytomegalovirus Positivity		
	**3 months and before**	**6 months and before**	**12 months and before**
**Follow-up for tuberculosis‡**			
Primary analysis			
>1 years of age			
CMV negative	1 (Referent)	1 (Referent)	1 (Referent)
Lower CMV values	2.0 (0.8-5.5)	3.7 (1.6-8.4)	2.7 (1.2-6.1)
Higher CMV values	3.7 (1.6-8.6)	3.6 (1.5-8.3)	3.7 (1.7-8.1)
P_{trend}	0.002	0.0035	0.0009
Tuberculosis episodes	31	33	37
Person-years, follow-up	4,882	5,028	5,138
>6 months of age			
CMV negative	1 (Referent)	1 (Referent)	1 (Referent)
Lower CMV values	1.8 (0.8-3.9)	2.8 (1.4-5.6)	2.4 (1.2-4.5)
Higher CMV values	2.8 (1.4-5.6)	3.4 (1.8-6.6)	3.1 (1.6-5.7)
P_{trend}	0.0031	0.0002	0.0004
Tuberculosis episodes	50	52	57
Person-years, follow-up	5,734	5,904	6,037
Secondary analyses			
1 to 2 years of age			
CMV negative	1 (Referent)	1 (Referent)	1 (Referent)
Lower CMV values	1.4 (0.2- 12.1)	4.9 (1.3-18.6)	3.9 (0.9-17.6)
Higher CMV values	6.6 (1.7-24.6)	3.2 (0.7- 14.3)	7.4 (1.9-28.9)
P_{trend}	0.0053	0.1361	0.0038

Tuberculosis episodes	10	12	14
Person-years, follow-up	1,664	1,713	1,753
2 to 9 years of age			
CMV negative	1 (Referent)	1 (Referent)	1 (Referent)
Lower CMV values	2.3 (0.7-7.1)	3.1 (1.0-8.9)	2.3 (0.8- 6.1)
Higher CMV values	2.6 (0.9-8.1)	3.8 (1.4-10.5)	2.5 (0.9-6.7)
P_{trend}	0.0905	0.0119	0.0711
Tuberculosis episodes	21	21	23
Person-years, follow-up	4,058	4,179	4,270
At any time point			
CMV negative	1 (Referent)	1 (Referent)	1 (Referent)
Lower CMV values	1.5 (0.5-3.0)	1.9 (1.0-3.5)	1.9 (1.1-3.4)
Higher CMV values	2.8 (1.5-5.0)	2.9 (1.7-4.9)	2.8 (1.6-4.7)
P_{trend}	0.0007	0.0003	0.0002
Tuberculosis episodes	66	69	74
Person-years, follow-up	5,739	5,910	6,042

* We did not include congenital cytomegalovirus (positive test <3 weeks of age) due to low statistical power.

‡ Follow-up time was restricted to certain ages based on distance from birth. The specified time indicates the starting point time. For example, the primary outcome is follow-up for tuberculosis starting at 1 year of age until the end of follow-up

† All models are adjusted for sex of the child, study site, and maternal HIV. Cox regression models were performed with differing timing of cytomegalovirus positivity (horizontal bars of table) and follow-up time for tuberculosis (left bars of table). Tests for linear trend across dose-response values (CMV negative, lower CMV values, high CMV values) were conducted after multivariable analysis and adjustment for other variables.

§ The majority of cytomegalovirus tests were performed prior to one year of age (N_{tests}=6,224; 89%).

5. Table S4. Sensitivity analyses showing adjusted hazard ratios with adjustment of tuberculin conversion through a censoring approach (main analysis) and not using a censoring approach (sensitivity analysis), by timing of cytomegalovirus positivity.

Model	3 weeks and before	6 weeks and before	3 months and before	6 months and before	12 months and before
**Follow-up for tuberculosis**					
**>1 year of age**	3.9 (0.9–16.4)	4.1 (1.2–13.8)	2.8 (1.4–5.8)	3.6 (1.7–7.3)	3.2 (1.6–6.4)
Total events	27	27	31	33	37
**Adjustment for:**					
Tuberculin conversion, main censoring approach	4.3 (1.0-19.1)	4.0 (1.2-13.9)	3.1 (1.4-6.7)	4.6 (2.0-10.5)	3.8 (1.7-8.4)
Total events	24	24	27	29	32
Tuberculin conversion, sensitivity analysis	4.0 (0.9-18.0)	3.5 (1.0-12.3)	3.0 (1.4-6.5)	4.5 (2.0-10.2)	3.7 (1.7-8.3)
Total events	24	24	27	29	32
**>6 months of age**	3.7 (1.1-11.9)	3.3 (1.2-9.3)	2.3 (1.3-4.1)	3.1 (1.8-5.5)	2.7 (1.6-4.6)
Total events	43	44	50	52	57
**Adjustment for:**					

Tuberculin conversion, main censoring approach	3.7 (1.1–12.3)	2.7 (1.0–7.9)	2.1 (1.2–3.8)	3.1 (1.7–5.8)	2.8 (1.5–5.1)
Total events	40	41	46	48	51
Tuberculin conversion, sensitivity analysis	3.3 (1.0–11.3)	2.1 (0.7–6.1)	2.0 (1.1–3.6)	2.9 (1.6–5.3)	2.6 (1.4–4.8)
Total events	40	41	46	51	51

6. Appendix Table S5. Sensitivity analyses showing adjusted hazard ratios with additional adjustment and exclusion of congenital cytomegalovirus infections, by timing of cytomegalovirus positivity.

Model	3 weeks and before	6 weeks and before	3 months and before	6 months and before	12 months and before
**Events**	27	27	31	33	37
**Primary model used in manuscript**	3.9 (0.9–16.4)	4.1 (1.2–13.8)	2.8 (1.4–5.8)	3.6 (1.7–7.3)	3.2 (1.6–6.4)
**Additional adjustment for:**					
Socioeconomic status summation score*‡	3.9 (0.9–16.6)	4.1 (1.2-13.8)	2.6 (1.3-5.5)	3.5 (1.7-7.3)	3.3 (1.6-6.5)
Socioeconomic status, groups‡	3.8 (0.9–16.2)	4.0 (1.2-13.5)	2.8 (1.4-5.8)	3.6 (1.8-7.3)	3.2 (1.6-6.4)
Household income, rand per month	3.8 (0.9–16.3)	4.1 (1.2-13.7)	2.8 (1.4-5.8)	3.6 (1.8-7.4)	3.3 (1.6-6.6)
Birthweight, grams*	4.0 (0.9-16.9)	3.8 (1.1-12.9)	2.7 (1.3-5.6)	3.3 (1.6-6.8)	3.0 (1.5-6.0)
Household tuberculosis exposure	4.3 (1.0-19.1)	4.0 (1.2-13.5)	2.8 (1.4-5.8)	3.7 (1.8-7.5)	3.2 (1.6-6.5)
Tuberculosis preventive therapy	3.8 (0.9–16.1)	3.8 (1.1-12.7)	2.8 (1.4-5.9)	3.6 (1.7-7.5)	3.1 (1.5-6.3)
Pneumonia event	3.8 (0.9–16.4)	3.9 (1.2-12.9)	2.4 (1.2-5.1)	3.1 (1.5-6.4)	2.7 (1.3-5.5)
Intensively sampled cohort	4.1 (1.2-13.6)	4.1 (1.2-13.6)	2.8 (1.4-5.8)	3.5 (1.7-7.2)	3.2 (1.6- 6.3)
**Exclusion:**					

Excluded congenital cytomegalovirus infections†	…	4.3 (0.7-31.9)	2.6 (1.2-5.6)	3.4 (1.6-7.0)	3.0 (1.5-6.2)

All models were adjusted for study site, maternal HIV, and child sex (in addition to specified variables on the left side of the table). The outcome was incident tuberculosis after one year of age.

† All congenital cytomegalovirus infections, defined as children with positive cytomegalovirus tests <3 weeks of age, were excluded from this analysis. This was performed to understand the impact of congenital cytomegalovirus to the relationship of subsequent tuberculosis development.

‡ Socioeconomic status comprised a comprehensive composite of asset ownership, household income, employment, and education.

7. Appendix Table S6. Multivariable mediation analyses† of tuberculin conversion and household tuberculosis exposure on the relationship between cytomegalovirus and risk of tuberculosis development.

Cytomegalovirus positivity at different time points	Multivariable model I: without tuberculin conversion or household exposure included	Multivariable model II: with tuberculin conversion* included	Multivariable model III: with household tuberculosis exposure included
**Follow-up for tuberculosis restricted to after 1 year of age**			
6 weeks and before	4.1 (1.2–13.8)	4.0 (1.2-13.9)	4.0 (1.2-13.5)
3 months and before	2.8 (1.4–5.8)	3.1 (1.4-6.7)	2.8 (1.4-5.8)
6 months and before	3.6 (1.7–7.3)	4.6 (2.0-10.5)	3.7 (1.8-7.5)
1 year and before	3.2 (1.6–6.4)	3.8 (1.7-8.4)	3.2 (1.6-6.5)
**Follow-up for tuberculosis not restricted**			
6 weeks and before	3.1 (1.2–7.8)	2.5 (1.0-6.4)	3.1 (1.2-7.8)
3 months and before	2.1 (1.3–3.4)	1.8 (1.1- 3.1)	2.1 (1.2-3.4)
6 months and before	2.3 (1.4–3.7)	2.2 (1.3-3.7)	2.3 (1.4-3.7)
1 year and before	2.3 (1.4–3.7)	2.4 (1.4-4.0)	2.3 (1.5-3.7)

Hazard ratios are displayed with 95% confidence intervals in parentheses and P values. Cox regression models were performed with differing timing of cytomegalovirus positivity (horizontal bars of table).

† All models are adjusted for sex of the child, study site, and maternal HIV and Multivariable model I is adjusted only for these three variables. Multivariable model II is adjusted only for all variables in Multivariable model I in addition to tuberculin conversion. Multivariable model III is adjusted only for all variables in Multivariable model I in addition to household tuberculosis exposure.

* Tuberculin conversion was restricted to conversions prior to one year of age.

8. Table S7. Cytomegalovirus positivity in the intensive and non-intensive cohort.

	Intensive cohort		Non-intensive cohort		All participants		P value
	n (%)	Total	n (%)	Total	n (%)	Total	
Timing of Cytomegalovirus Positivity							
3 weeks and before	17 (2.3)	752	1 (1.6)	61	18 (2.2)	813	0.751
6 weeks and before	26 (3.4)	760	1 (1.6)	62	27 (3.3)	822	0.442
3 months and before	162 (20.5)	792	21 (24.1)	87	183 (20.8)	879	0.422
6 months and before	280 (34.7)	806	35 (35.0)	100	315 (34.8)	906	0.959
12 months and before	340 (41.6)	817	47 (42.0)	112	387 (41.7)	929	0.944
24 months and before	384 (47.0)	817	51 (45.5)	112	435 (46.8)	929	0.771

9. Table S8. Population attributable fractions of tuberculosis disease at distinct time points due to cytomegalovirus at corresponding time points.

Timing of Cytomegalovirus Positivity	Follow-up for tuberculosis	CMV positive	N	Prevalence of exposure	AHR (95% CI)	PAF (95% CI)
6 months and before	>6 months of age	315	906	34.8	3.1 (1.8-5.5)	42.2 (21.8–61.0)
12 months and before	>12 months of age	387	929	41.7	3.2 (1.6–6.4)	47.9 (20.5–69.3)
24 months and before	>24 months of age	435	929	46.8	3.0 (1.2-7.2)	48.3 (8.9–74.4)

10. Table S9. P-value tests of nonzero slope in a generalized linear regression of the scaled Schoenfeld residuals on time

	Timing of Cytomegalovirus Positivity					
	**3 weeks and before***	**6 weeks and before**	**3 months and before**	**6 months and before**	**12 months and before**	**24 months and before§**
**Follow-up for tuberculosis‡**						
**Primary analysis**						
>6 months of age	0.8851	0.8044	0.8001	0.8021	0.8193	0.8146
>1 years of age	0.855	0.8467	0.965	0.968	0.9732	0.9779
**Secondary analyses**						
6 months to 1 year	0.3837	0.2724	0.2538	0.4628	0.4505	0.4409
1 to 2 years of age	0.5553	0.5441	0.6118	0.5339	0.8841	0.6164
2 to 9 years of age	0.855	0.8467	0.965	0.968	0.9732	0.9779
At any time point	0.7669	0.6959	0.5328	0.3648	0.616	0.5906

We tested the proportional-hazards assumption on the basis of Schoenfeld residuals after fitting each Cox model. More specifically, we evaluated the proportional hazards assumption by tests of nonzero slope in a generalized linear regression of the scaled Schoenfeld residuals on time

(Grambsch and Therneau, 1994). We tested the null hypothesis of zero slope, which is equivalent to testing that the log hazard-ratio function is constant over time. Rejection of the null hypothesis of a zero slope signifies that the proportional-hazards assumption was not met.

We tested the proportional hazards assumption in each Cox model.

11. Table S10. Median cytomegalovirus load and categorization cutoffs of low versus high viral loads

	**Median (IQR) viral load among CMV positives, viral load copies per ml**	**Median (IQR) viral load among CMV positives, log10 copies per ml**	**Low versus high viral load categorization, log10 copies per ml**
Timing of Cytomegalovirus Positivity			
3 months and before	21,989.7 (4,778.4, 96,473.8)	10.0 (8.5, 11.5)	10.0
6 months and before	35,125.0 (6,097.2, 12,9239.1)	10.8 (9.0, 12.1)	10.8
12 months and before	66,087.3 (14,832.21, 214,049.2)	11.1 (9.6-12.3)	11.1
24 months and before	56,863.8 (11,636.1, 19,3019.5)	10.9 (9.4, 12.2)	10.9

12. Table S11. Timing of cytomegalovirus positivity and breastfeeding.

Timing of Cytomegalovirus Positivity	Breastfed	Not Breastfed	P Value‡
3 weeks and before			0.213
Yes	15 (2.0)	3 (4.1)	
No	727 (98.0)	70 (95.9)	
6 weeks and before			0.09
Yes	22 (2.9)	5 (6.8)	
No	728 (97.1)	69 (93.2)	
3 months and before			0.004
Yes	177 (22.0)	6 (8.0)	
No	629 (78.0)	69 (92.0)	
6 months and before			<0.0001
Yes	304 (36.6)	11 (14.3)	
No	527 (63.4)	66 (85.7)	
12 months and before			<0.0001
Yes	375 (44.0)	11 (14.3)	
No	478 (56.0)	66 (85.7)	
24 months and before§			<0.0001
Yes	428 (48.9)	16 (20.8)	
No	448 (51.1)	61 (79.2)	

‡ Exact or chi-square P-values were used as appropriate.

§ The majority of cytomegalovirus tests were performed prior to one year of age (N_{tests}=6,224; 89%).

13. Table S12. Children diagnosed with tuberculosis and CMV positivity at a given time point.

	**Diagnosed tuberculosis at some point in the study**
Timing of Cytomegalovirus Positivity	
3 weeks and before*	
Yes	3
No	56
6 weeks and before	
Yes	5
No	57
3 months and before	
Yes	24
No	46
6 months and before	
Yes	40
No	34
12 months and before	
Yes	48
No	31
24 months and before§	
Yes	56
No	24

14. Table S13. Cytomegalovirus status based on the timing of cytomegalovirus testing and the study site.

	**Mbekweni**		**TC Newman**		**P value**
	**n (%)**	**Total**	**n (%)**	**Total**	
Timing of Cytomegalovirus Positivity					
3 weeks and before	13 (2.4)	534	5 (1.8)	281	0.545
6 weeks and before	19 (3.5)	540	8 (2.8)	284	0.591
3 months and before	106 (18.9)	562	77 (24.2)	319	0.064
6 months and before	189 (33.1)	571	126 (37.4)	337	0.19
12 months and before	229 (39.6)	579	157 (44.7)	351	0.12
24 months and before	263 (45.3)	580	181 (48.5)	373	0.337

15. Table S14. STROBE Statement.

	Item No	Recommendation	Page No
**Title and abstract**	1	(*a*) Indicate the study's design with a commonly used term in the title or the abstract	1
		(*b*) Provide in the abstract an informative and balanced summary of what was done and what was found	4
**Introduction**			
Background/rationale	2	Explain the scientific background and rationale for the investigation being reported	6
Objectives	3	State specific objectives, including any prespecified hypotheses	6
**Methods**			
Study design	4	Present key elements of study design early in the paper	7
Setting	5	Describe the setting, locations, and relevant dates, including periods of recruitment, exposure, follow-up, and data collection	7
Participants	6	(*a*) Give the eligibility criteria, and the sources and methods of selection of participants. Describe methods of follow-up	7-8
		(*b*) For matched studies, give matching criteria and number of exposed and unexposed	N/A
Variables	7	Clearly define all outcomes, exposures, predictors, potential confounders, and effect modifiers. Give diagnostic criteria, if applicable	7-10
Data sources/ measurement	8*	For each variable of interest, give sources of data and details of methods of assessment (measurement). Describe comparability of assessment methods if there is more than one group	7-10
Bias	9	Describe any efforts to address potential sources of bias	8-10
Study size	10	Explain how the study size was arrived at	7
Quantitative variables	11	Explain how quantitative variables were handled in the analyses. If applicable, describe which groupings were chosen and why	8-10
Statistical methods	12	(*a*) Describe all statistical methods, including those used to control for confounding	9-10
		(*b*) Describe any methods used to examine subgroups and interactions	
		(*c*) Explain how missing data were addressed	
		(*d*) If applicable, explain how loss to follow-up was addressed	
		(*e*) Describe any sensitivity analyses	
**Results**			

Participants	13*	(a) Report numbers of individuals at each stage of study—eg numbers potentially eligible, examined for eligibility, confirmed eligible, included in the study, completing follow-up, and analysed<br>(b) Give reasons for non-participation at each stage<br>(c) Consider use of a flow diagram	12
Descriptive data	14*	(a) Give characteristics of study participants (eg demographic, clinical, social) and information on exposures and potential confounders<br>(b) Indicate number of participants with missing data for each variable of interest<br>(c) Summarise follow-up time (eg, average and total amount)	12
Outcome data	15*	Report numbers of outcome events or summary measures over time	12-13
Main results	16	(*a*) Give unadjusted estimates and, if applicable, confounder-adjusted estimates and their precision (eg, 95% confidence interval). Make clear which confounders were adjusted for and why they were included<br>(*b*) Report category boundaries when continuous variables were categorized<br>(*c*) If relevant, consider translating estimates of relative risk into absolute risk for a meaningful time period	13-14
Other analyses	17	Report other analyses done—eg analyses of subgroups and interactions, and sensitivity analyses	13-14
**Discussion**			
Key results	18	Summarise key results with reference to study objectives	15
Limitations	19	Discuss limitations of the study, taking into account sources of potential bias or imprecision. Discuss both direction and magnitude of any potential bias	17
Interpretation	20	Give a cautious overall interpretation of results considering objectives, limitations, multiplicity of analyses, results from similar studies, and other relevant evidence	15-17
Generalisability	21	Discuss the generalisability (external validity) of the study results	15-16
**Other information**			
Funding	22	Give the source of funding and the role of the funders for the present study and, if applicable, for the original study on which the present article is based	23

16. Table S15. Number of censored participants in the primary and sensitivity analyses.

Characteristics	1st TST	2nd TST	3rd TST	4th TST	5th TST	6th TST
**Primary analysis**						
N	812	348	152	56	23	…
N positive (≥10 mm)	91	24	9	5	0	…
Proportion positive	0.112	0.069	0.059	0.089	0	…
Median age, months (IQR)	6.0 (5.9-10.6)	12.0 (10.5-24.0)	23.7 (15.0-35.0)	23.9 (17.5-34.9)	24.6 (18.1-35.5)	…
Mean age, months (SD)	9.1 (7.7)	17.5 (11.2)	24.5 (11.6)	25.4 (11.6)	26.7 (12.2)	…
Median TST induration (IQR)	0 (0-4)	0 (0-2)	0 (0-0)	0 (0-0)	0 (0-0)	…
Mean TST induration (IQR)	2.8 (4.8)	2.4 (5.1)	1.1 (3.8)	1.4 (3.9)	0 (0)	…
N censored due to reactive, negative skin test	228	34	6	3	0	…
**Sensitivity analysis, no censoring of participants with reactive but negative tests**						
N	812	522	256	104	42	17
N positive (≥10 mm)	91	54	15	4	3	0
Proportion positive	0.112	0.103	0.059	0.038	0.071	0
Median age, months (IQR)	6.0 (5.9-10.6)	12.0 (10.5-24.0)	23.9 (14.2-35.1)	26.2 (20.6-36.6)	28.7 (20.1-35.6)	25.3 (17.0, 40.4)
Mean age, months (SD)	9.1 (7.7)	17.4 (11.1)	24.8 (11.6)	28.7 (12.0)	28.2 (12.1)	28.8 (13.7)
Median TST induration (IQR)	0 (0-4)	0 (0-0)	0 (0-0)	0 (0-0)	0 (0-0)	0 (0-0)
Mean TST induration (IQR)	2.8 (4.8)	2.2 (4.9)	1.1 (3.6)	1.0 (3.1)	1.5 (5.2)	0.3 (1.2)

17. Table S16. Tuberculin skin test indurations among children acquiring and not acquiring cytomegalovirus.

	Median TST induration	Mean TST induration	P Value
			0.31
CMV Positive by 3 weeks	0 (0-4)	2.9 (5.1)	
CMV Positive by 3 weeks	0 (0-4)	2.8 (4.9)	
			0.83
CMV Positive by 6 weeks	0 (0-3)	1.4 (3.9)	
CMV Negative by 6 weeks	0 (0-4)	1.6 (4.9)	
			0.9454
CMV Positive by 3 months	0 (0-4)	2.9 (5.1)	
CMV Negative by 3 months	0 (0-4)	2.9 (4.8)	
			0.6266
CMV Positive by 6 months	0 (0-4)	3.0 (5.1)	
CMV Negative by 6 months	0 (0-4)	2.7 (4.7)	
			0.95
CMV Positive by 1 year	0 (0-4)	2.5 (5.0)	
CMV Negative by 1 year	0 (0-4)	2.7 (4.8)	

Tuberculin skin test indurations were compared among children with and without cytomegalovirus infection at different age timepoints using Mann–Whitney U tests.
